# Sinking flux of particulate organic matter in the oceans: Sensitivity to particle characteristics

**DOI:** 10.1038/s41598-020-60424-5

**Published:** 2020-03-27

**Authors:** Melissa M. Omand, Rama Govindarajan, Jing He, Amala Mahadevan

**Affiliations:** 1 University of Rhode Island, Graduate School of Oceanography, Narragansett, RI 02882 USA; 2 International Centre for Theoretical Sciences, Tata Institute of Fundamental Research, Bangalore, 560089 India; 3 Massachusetts Institute of Technology, Cambridge, MA 02139 USA; 4 Woods Hole Oceanographic Institution, Woods Hole, MA 02543 USA

**Keywords:** Biogeochemistry, Physical oceanography

## Abstract

The sinking of organic particles produced in the upper sunlit layers of the ocean forms an important limb of the oceanic biological pump, which impacts the sequestration of carbon and resupply of nutrients in the mesopelagic ocean. Particles raining out from the upper ocean undergo remineralization by bacteria colonized on their surface and interior, leading to an attenuation in the sinking flux of organic matter with depth. Here, we formulate a mechanistic model for the depth-dependent, sinking, particulate mass flux constituted by a range of sinking, remineralizing particles. Like previous studies, we find that the model does not achieve the characteristic ‘Martin curve’ flux profile with a single type of particle, but instead requires a distribution of particle sizes and/or properties. We consider various functional forms of remineralization appropriate for solid/compact particles, and aggregates with an anoxic or oxic interior. We explore the sensitivity of the shape of the flux vs. depth profile to the choice of remineralization function, relative particle density, particle size distribution, and water column density stratification, and find that neither a power-law nor exponential function provides a definitively superior fit to the modeled profiles. The profiles are also sensitive to the time history of the particle source. Varying surface particle size distribution (via the slope of the particle number spectrum) over 3 days to represent a transient phytoplankton bloom results in transient subsurface maxima or pulses in the sinking mass flux. This work contributes to a growing body of mechanistic export flux models that offer scope to incorporate underlying dynamical and biological processes into global carbon cycle models.

## Introduction

**Figure 1:**
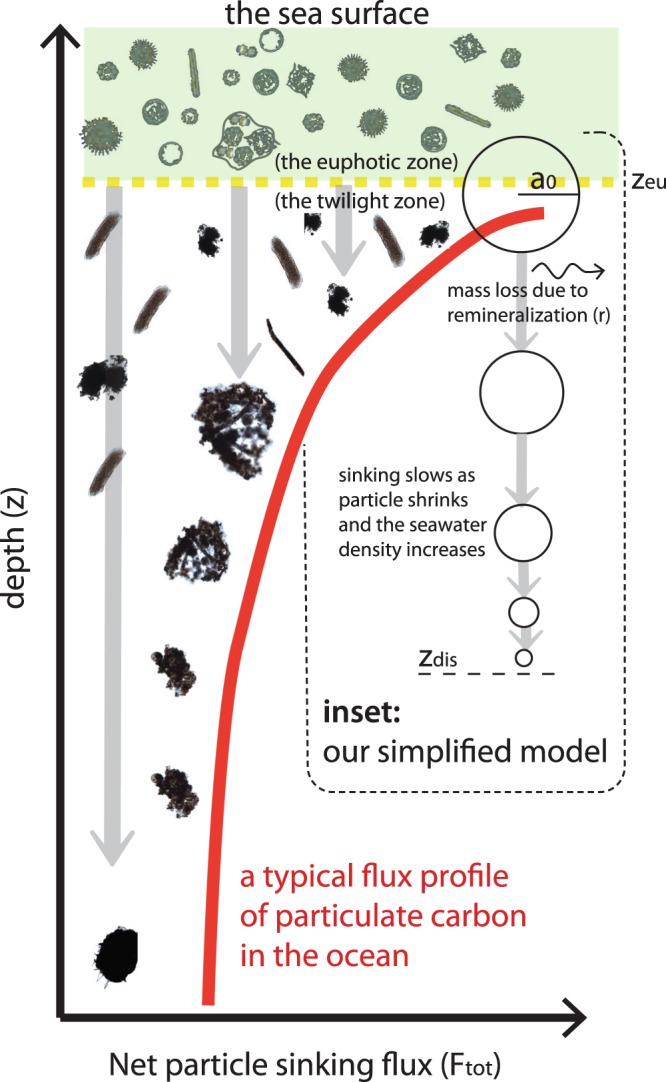
Though sinking oceanic particles (inset images) encompass a wide range of shape, porosity, ballast and other characteristics, our model attempts to capture some of the predominant features that influence the shape of the sinking flux profile (red line). Our simplified model is depicted (inset); the spheres represent either solid particles or aggregates. These particles (initial radius a_0_), produced within the sunlit euphotic zone (green region extending to z_eu_), sink at a rate predicted by Stokes law. They slow as they reach greater depths due to their shrinking volume and increasing water density and would entirely disappear at z_dis_. The images of inset particles are sinking phytoplankton, pellets and aggregates captured in particle-preserving gel traps by Dr. Colleen Durkin.

The oceans absorb about 2.5 × 10^12^ kg of carbon from the atmosphere annually, amounting to a net uptake of about 40% of the anthropogenically produced CO_2_ since industrialization^1^. Understanding the controls on the oceanic uptake of atmospheric CO_2_ is important for predicting how the uptake rate might change in the future. The oceanic biological pump^2^ plays a significant role in this process: Phytoplankton produce organic matter by taking up dissolved inorganic carbon through photosynthesis in the sunlit ocean. While a major portion of this production is recycled, a small fraction (typically, less than 20%) rains out to depth, carrying with it organic carbon, nutrients and minerals. Such particles are formed through a variety of mechanisms: cells age, form cysts, are ingested and egested by zooplankton, aggregate, coagulate, and gradually sink as ‘marine snow’. The sinking material varies in shape, size and character, ranging from individual cells to pellets and aggregates, most of which is rapidly colonized and consumed by heterotrophic bacteria, contributing to the attenuation of the sinking flux with depth (Fig. 1).

The shape of the depth-diminishing flux profile has traditionally been characterized by empirical fits to data obtained from particle intercepting sediment traps^3^ or radionuclide disequlibria^4^. A commonly used form of the flux profile is the “Martin curve”^5^, which describes the attenuating, sinking flux by a power law $F(z)=F({z}_{eu}){(z/{z}_{eu})}^{-0.858}$, fit from flux measurements at different depths in the ocean. Here, the euphotic depth, *z*_*eu*_, is the depth by which 99% of the incoming shortwave radiation is attenuated, and *F*(*z*_*eu*_) is the flux leaving the euphotic layer, within which most phytoplankton is produced and grazed. Other forms of the flux profile include the form^3^ *F*(*z*) = *P*_*s*_/(*az* + *b*), where *P*_*s*_ the net primary production of particles at the surface and *a*, *b* are constants derived from data, and a double exponential form^6^ that accommodates two carbon pools for labile and refractory fractions.

Models of the oceanic carbon cycle use such a flux profile to prescribe the redistribution of carbon and nitrogen with depth in terms of the overlying production. It is often characterized by the ‘export efficiency’ or the fraction of export that reaches a depth of 100 m. The shape of the profile impacts the redistribution of nutrients and biogeochemical properties in the ocean. Though it has been demonstrated that global carbon models are sensitive to the flux profile shape^7^, the computational expense of incorporating mechanistic particle fluxes into global circulation models (GCMs) has largely precluded their inclusion. Most use fixed forms of the flux profile that do not account for the regional and temporal variability in the nature of particulate organic matter (POM) and its remineralization rates.

Several previous studies^8–11^ have formulated prognostic models for the sinking particle flux. Kriest and Oschiles^12^ examined what sinking characteristics contribute to a ‘Martin curve’ like flux profile and showed that a constant sinking velocity does not produce the characteristic attenuation in the profile. DeVries *et al*.^10^, proposed a model called PRiSM and constrained the model parameters to provide an optimal fit to the global phosphate distribution, thereby showing that the flux profile needs to be spatially variable to be consistent with oceanic dissolved nutrients. They implemented the particle sinking model with remineralization processes in a global circulation model with advection to obtain regionally variable flux profiles. Here, we formulate a mathematical model for sinking, remineralizing particles to give a spatially and temporally varying estimate of their cumulative sinking flux. Our model is similar to DeVries *et al*.^10^, but our approach differs in that we model the time-dependent, sinking depth and volume of each size class of particles – and then sum over all size classes to derive a cumulative flux profile. We find this approach instructive in that it allows us to examine the contribution of specific size classes. Hence, we present a derivation of the model, even though the steady-state aggregate flux profile solution is similar to what was proposed in previous studies^10,13^.

Our model makes several assumptions – it assumes a Stokes’ sinking velocity and neglects particle interactions, aggregation and breakup. We use a simplified fractal scaling to account for complexity of particle structures and shapes, which leads to deviations in particle behavior from that of the ideal spherical particle. In comparison, DeVries *et al*.^10^ use a modified power law to describe the sinking velocity and dependence of mass on the particle dimension. Our model also considers the role of density stratification in the ocean. Further, we present a generalized functional form for remineralization and discuss the circumstances under which one form may be more appropriate than another. We examine how the functional form of remineralization affects the transfer efficiency and flux, which was not addressed in previous studies. We also examine how the time-variability of particle characteristics produced, for example, by community shifts in the ecosystem, influences the transient shape of the flux profile.

The four primary parameters we consider in our model are particle density, particle remineralization rate, the initial particle size distribution (PSD) of material exiting the euphotic zone, and the water column density gradient. We show that different combinations of these parameters result in markedly different shapes in the flux profile. Similar to Cram *et al*.^13^, we find the ‘transfer efficiency’ to be highly sensitive to the remineralization rate and the particle size spectrum. Cram *et al*.^13^ found their model was less sensitive to the dissolved oxygen concentration – a factor we did not include in ours. Though the simple model presented here does not account for many complex biological processes and characteristics that affect the particles and their sinking flux in the oceans, we believe that such models provide a useful framework for examining the relative importance of various parameters and will help understand some of the observed variability in export flux observations. An advantage of simple prognostic models^10^ is that they provide an analytical solution that can readily be incorporated into a General Circulation Model (GCM) with little computational expense to account for the effects of advection on the particle flux.

## Results

### Preliminaries: A single, sinking, remineralizing particle

We start by considering a single particle, sinking vertically through the ocean at its terminal velocity, *w*_*sink*_. Several early experiments on oceanic particles attempted to characterize sinking rates^14^ using Stokes’ Law^15,16^. While departures from Stokes’ theory result from particle shapes deviating from spherical, variations in structural from, porosity, rigidity and drag^17^, an assumption of Stokes’ Law is frequently applied to modern flux parameterizations^10,18–21^ and is used here with cognizance of its limitations.

For a particle of radius 1 mm, a typical sinking speed of 100 m d^−1^, and the kinematic viscosity of water at room temperature, the particle Reynolds number is about 1. At low particle Reynolds number, and making the reasonable assumption that particle sinking is in quasi steady state, individual spherical particles have a sinking velocity *w*_*sink*_, described as the balance between the downward gravitational force and the upward Stokes’ drag as

1 $${w}_{sink}=\frac{2{a}^{2}g({\rho }_{p}-{\rho }_{f})}{9\mu },$$

where *a* is the effective particle radius, *μ* is the dynamic viscosity of the fluid, *g* is the acceleration due to gravity, and *ρ*_*p*_ and *ρ*_*f*_ are the densities of the particle and surrounding fluid, respectively. The kinematic viscosity *μ* varies between 0.8 × 10^−3^ and 1.7 × 10^−3^ kg m^−1^s^−1^ derived from the range of typical temperature and salinity observed throughout the global ocean^22^. For this analysis we use *μ* = 1 × 10^−3^ kg m^−1^s^−1^ The characterization of particle size, sinking and remineralization in terms of an effective radius *a* is an assumption that permeates through the development of this model. While it is recognized that a majority of marine particles are non-spherical, such a characterization facilitates quantification and a simple analytic form. To treat a greater variety of particle shapes, we later use the fractal dimension as a way to account for complex particles.

Our model assumes that particles are colonized by bacteria upon leaving the base of the euphotic zone and are consumed steadily thereafter. At first order, the depth to which a particle will sink is therefore a balance between its sinking velocity and the rate at which it is remineralized *r* (per day, expressed as d^−1^). This rate depends on several factors including, but not limited to, the particle constituents, shape, porosity and surface area available for bacterial colonization^23^, the ambient temperature^24^, microbial respiration rates^25^, and can vary in time and space^26^. For this study we use a range reported in Iversen *et al*.^27,28^, with an average carbon-specific respiration rate of individual aggregates *r* = 0.12 ± 0.03 d^−1^ in a 15 °C treatment, and *r* = 0.03 ± 0.01 d^−1^ in a 4 °C treatment, corresponding to a life-span (*r*^−1^) of approximately 8 days and 33 days, respectively.

The functional form of bacterial solubilization likely depends on the particle surface area available, the degree of colonization and hence the degree of anoxia within a sinking particle, among other ambient factors such as temperature and oxygen concentration^13^. We propose a generalized form *dV*∕*dt* ~ − *ra*^*n*^, which allows the particle volume *V* (and thus, mass) to diminish with time in a few ways: (a) at a constant rate (*n* = 0), suggesting that there is a fixed pace of remineralization irrespective of particle size, (b) proportional to the surface area (*n* = 2) suggesting that only the external surface of a (roundish) particle is being remineralized and that the number of bacteria (and thus the rate) decrease as the available surface area is reduced (likely appropriate for a very compact particle, or one that has an anoxic interior), or (c) proportional to the particle volume (or mass) (*n* = 3) suggesting that the bacteria have colonized the interior and exterior of the particle (likely appropriate for aggregates that are more like a sponge than a compact sphere). The case (c) was considered by DeVries *et al*.^10^ and Cram *et al*.^13^. The generalized expression for the rate of change of the particle radius is:

2 $$\frac{da}{dt}=-\,Cr{a}^{(n-2)},$$

where *C* is a constant that relates to the form of remineralization. For the following derivation, we use the intermediate case *n* = 2.

We relate the particle density *ρ*_*p*_ to the fluid density at the base of the euphotic zone (*ρ*_*T*_) using a dimensionless factor *α* as

3 $${\rho }_{p}={\rho }_{T}(1+\alpha ).$$

The range of *α* we anticipate based on excess density observations from the literature is reported in Table 1. Assuming a linear change in density with depth *β* (units *m*^−1^), and assuming incompressible particles (see the section Particle Compressibility), we write the fluid density *ρ*_*f*_ at any depth *z* in terms of *ρ*_*T*_ as

4 $${\rho }_{f}(z)={\rho }_{T}(1+\beta z).$$

Our model parameters thus far are *α*, *β* and remineralization rate *r*. Substituting 4 and 3 into 1, and using the *n* = 2 remineralization equation, the depth- and time-dependent particle sinking velocity becomes

5 $${w}_{sink}=\frac{2\ g\ {\rho }_{T}\ {a}_{o}^{2}}{9\ \mu }(\alpha -\beta z){(1-rt)}^{2}.$$

Integrating from the initial particle location at the base of the euphotic zone, taken to be *z* = 0 at time *t* = 0, to some depth *z* and time *t*, we arrive at the solution for the particle depth as a function of its volume $V(t)=\frac{4}{3}\pi {[{a}_{0}(1-rt)]}^{3}$ as

6 $$z(t)=\frac{\alpha }{\beta }(1-{e}^{\gamma [V(t)-{V}_{0}]}),\,{\rm{w}}{\rm{h}}{\rm{e}}{\rm{r}}{\rm{e}}\,\gamma =\frac{{\rho }_{T}\beta g}{18\pi \mu r{a}_{0}},\,{\rm{a}}{\rm{n}}{\rm{d}}\,{V}_{0}\,{\rm{i}}{\rm{s}}\,{\rm{t}}{\rm{h}}{\rm{e}}\,{\rm{i}}{\rm{n}}{\rm{i}}{\rm{t}}{\rm{i}}{\rm{a}}{\rm{l}}\,{\rm{v}}{\rm{o}}{\rm{l}}{\rm{u}}{\rm{m}}{\rm{e}}.$$

The particle volume, as a function of depth, is therefore

7 $$V(z)=\frac{1}{\gamma }{\rm{l}}{\rm{o}}{\rm{g}}\,\left(1-\frac{\beta }{\alpha }z\right)+{V}_{0}.$$

**Table 1:** Summary of observations of excess particle density *ρ*_*p*_ − *ρ*_*f*_, remineralization rate *r*, and number spectral slope *p* used to define the range of our model parameters.

	*observed range*	*particle description*	*citation*
*ρ*_*p*_-*ρ*_*f*_ [kg m ^−3^]	10–30	living diatoms	^47^
	40 to 80	senescent diatoms	^47^
	30 to 500	ESD 1–362 *μ*m	^29^
	2 to 0.05	aggregates	^40^
*r* [d^−1^]	0.02 to 0.2	labile material	^49^
	0.003 to 0.02	non-labile material	^49^
	0.26	PON (18 °C)	^49^
	0.04	PON (8 °C)	^49^
	0.13 ± 0.07	aggregates	^27^
	0.12 ± 0.03	aggregates (15 °C)	^28^
	0.03 ± 0.01	aggregates (4 °C)	^28^
number spectral slope *p*	−2.4 to −3.6	ESD 1–100 *μ*m	^29^
	−3.0	ESD 20–3000 *μ*m	^33^
	−2.5 to −4.5	ESD > 100 *μ*m	^19^
	−2.2 to −3.3	ESD 0.15–6.5 mm	^27^

**Figure 2:**
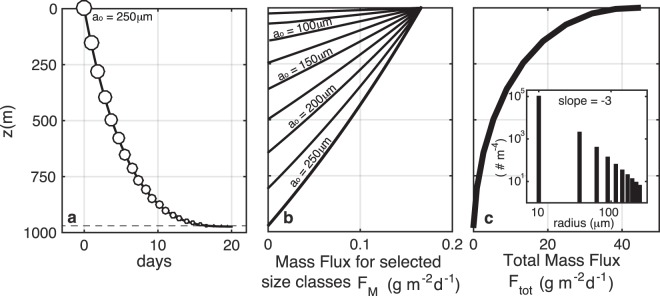
(**a**) A particle (*a*_*o*_ = 250 *μ*m, *α* = 0.03) slows as it sinks into denser water (*β* = 5 × 10^−6^ m^−1^) and is remineralized (*r* = 0.11 d^−1^), until disappearing at *z*_*dis*_ = 980 m (Eq. 8). (**b**) Mass flux profiles (Eqn 9, given the initial size spectrum shown in the inset of panel c, and *N*_*o*_ = 10^4^ particles m^−2^d^−1^) for particle size classes ranging from *a*_*o*_ = 10 to 250 *μ*m. (**c**) The total mass flux (eqn 11). Note that for our model, *z* = 0 represents the base of the euphotic zone.

As conceptualized, (6) predicts that the particle descends quickly at first, but slows as it is remineralized and as the relative difference between the particle and fluid density diminishes (Fig. 2a). The particle eventually disappears as *V*(*z*) approaches zero (dashed line in Fig. 2a) at depth

8 $${z}_{dis}=\frac{\alpha }{\beta }(1-{e}^{-\gamma {V}_{0}}).$$

### Sinking flux for particles of single size

The total particle flux is the sum of a population of particles all undergoing a similar fate. In steady state, for particles of initial radius *a*_*o*_ raining down from *z* = 0 at a rate of *N*_*o*_ particles per m^2^ per day, the depth-dependent mass flux *F*_*M*_(*z*, *a*_0_) is the product of *N*_*o*_ (which is depth independent under the assumption of steady state) and the mass (*M*) of an individual particle at depth *z* given by *M*(*z*) = *ρ*_*p*_*V*(*z*). This is valid until the particle disappears, and hence

9 $${F}_{M}(z,{a}_{0})=\left\{\begin{array}{ll}{N}_{0}\ {\rho }_{p}\left[\frac{1}{\gamma }log\left(1-\frac{\beta }{\alpha }z\right)+{V}_{0}\right] & {\rm{f}}{\rm{o}}{\rm{r}}\ z\le {z}_{dis}\\ 0 & {\rm{f}}{\rm{o}}{\rm{r}}\ z > {z}_{dis}.\end{array}\right.$$

Two relevant depths can be derived from these equations: the disappearing depth (*z*_*dis*_) given by eqn (8) and the suspension depth (${z}_{susp}=\frac{\alpha }{\beta }$) where a particle becomes neutrally buoyant. We find that under typical oceanic conditions and particle traits listed in Table 1, *z*_*susp*_ ≫ *z*_*dis*_, and so particles are far more likely to be remineralized before reaching a neutral density. By extension, for most *z*≤*z*_*dis*_, the term $\frac{\beta }{\alpha }z\ll 1$. Hence, by Taylor expansion, the mass flux is, to good approximation, a linearly decreasing function of depth (as seen in Fig. 2b)

10 $${F}_{M}(z,{a}_{0})\approx {N}_{0}\ {\rho }_{p}\left(-\frac{\beta }{\gamma \alpha }z+{V}_{0}\right)\ {\rm{f}}{\rm{o}}{\rm{r}}\ z\le {z}_{dis}.$$

This solution demonstrates that a single size class of identical particles does not reproduce the canonical Martin curve flux profile (Fig. 2b). We show in the following section, that a range of particle sizes (or types) is required to generate the canonical concave downward flux profile.

### A range of particle sizes and types

Among the most widely reported characteristics of oceanic particles is the vast abundance of small particles relative to larger particles^19,29^. When plotted on a logarithmic scale, the particle number spectrum has a negative slope *p* (the ‘number spectral slope’), suggesting that particle sizes are distributed according to a power-law^18,30–33^. This slope has been observed to vary widely, regionally, in time, and with depth, generally ranging between *p* = −4 and *p* = −2 (Table 1)^19^. Since the volume (and mass) of particles scales as *M* ~ *a*^3^ for spherical particles, a steep slope (*p* < −3) indicates a dominance of small particles in the mass flux, whereas a flatter slope (*p* > − 3) suggests that larger particles will dominate the mass flux.

**Figure 3:**
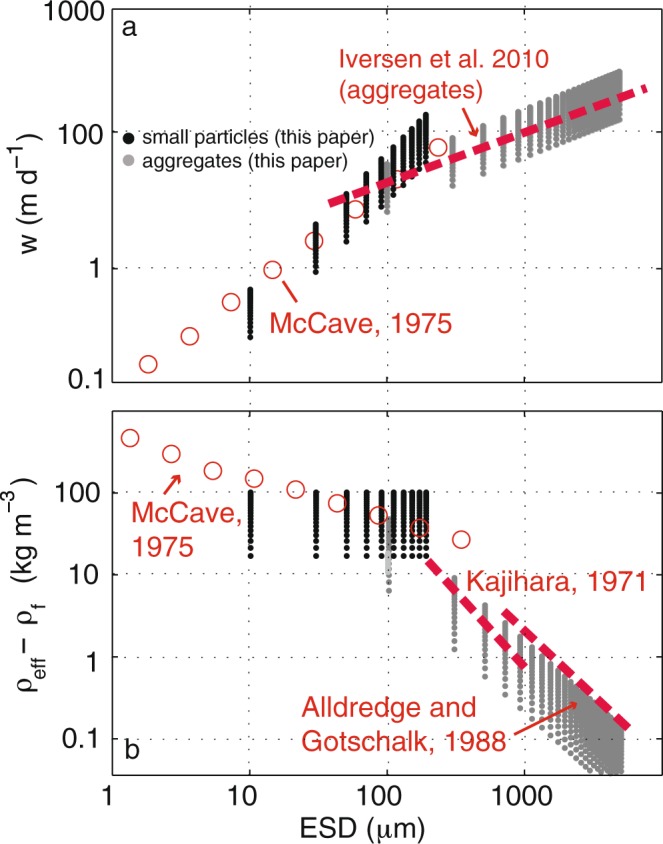
(**a**) Initial modeled particle sinking velocity (m d^−1^) and (**b**) excess density (*ρ*_*eff*_ − *ρ*_*T*_) as a function of size (equivalent spherical diameter ESD) for small particles (*ρ*_*p*_ = *ρ*_*eff*_, black points) and aggregates (gray points) at *t*=0 released at the base of the euphotic zone. The fluid density *ρ*_*T*_ = 1028 kg m^−3^ and *α* ranges from 0.01 to 0.1. A fractal dimension of 1.85 and *A*_*f*_ = 1.49^38^ reproduces the flattened *w* spectrum observed in situ for larger, fluffier aggregates (red dashed lines) with less excess density^27,39,40^.

We define a range of initial size classes *a*_*o*,*i*_ that have a power law distribution such that the number flux is ${N}_{i}({a}_{i}) \sim {a}_{i}^{-p}$ particles m^−2^day^−1^. The total flux at depth *z* is the linear sum (or integral) of the mass flux in each size class (described by eqn 10) according to

11 $${F}_{tot}(z)=\mathop{\sum }\limits_{i=0}^{{n}_{max}}{F}_{M}^{i}(z,{a}_{i}),$$

which produces a total mass flux that attenuates with depth in a manner consistent with the canonical flux profile (Fig. 2c). An important aspect of the summation is that particles in the initial size classes *a*_*i*_ cease to contribute to the flux profile beneath the depths *z*_*dis*_(*a*_*i*_) at which they become extinct. We can also devise a scenario in which particles are the same size, but differ in their mineral composition or source of origin, which could result in a distribution of *r* or *α*. Applying log-normal distributions of *r* or *α* to particles within one size class also produces flux profiles (not shown) that qualitatively resemble the observed shape. Since the particles are remineralized with depth, and each size class ceases to exist beyond its own *z*_*dis*_, the size spectral slope of the particles changes with depth. The smallest initial size classes are remineralized nearer the surface, and the larger sizes transition to smaller-size categories. Thus the spectrum of the number density (PSD) of raining particles flattens with depth (Supplementary Sec. 1, Fig. S1a,b). The spectrum of mass flux (Supplementary Fig. S1c,d) for the case with a number spectral slope *p* = −3 is initially flat, and steepens with depth, as the particles that started small disappear altogether, and the particles that started large become smaller. While some observations [cf. Fig. 7, Laurenceau-Cornec *et al*.^34^] are suggestive of the predictions made here, the high variability in others [cf. Fig. 2, Guidi *et al*.^19^], precludes conclusive evidence that a flattening of the PSD occurs with depth in the global oceans. Similar models that use a Stokes’ sinking rate^10^ also predict a flattening of the spectrum, while more recent approaches^35^ have incorporated a disaggregation term to restore the small particle class. Alternatively, small particles may be more persistent at depth because they are sinking more rapidly than predicted by Stokes’ model^36,37^, perhaps due to a different lability, density (ballast), or fractal dimension than larger aggregate particles. Thus, we consider two types of particle: a small ‘solid’ particle whose effective density is independent of size, and aggregates that have an effective density that decreases with increasing size (Fig. 3b).

### Aggregates

For large particles, numerous observations of settling rate versus size demonstrate that the spectrum is flatter than that predicted by Stokes’ equation applied to spherical particles^9,27^. Abundant observations from in situ cameras^19^, laser imaging^41^, SCUBA divers^40^, and gel traps indicate much of the particle mass in the twilight zone is composed of ‘marine snow’, aggregates, or clumps of smaller particles that form ‘fluffy’ particles up to 5 ~ mm^27^. Compared to a solid particle of similar size and shape, the reduction in the effective density of these porous particles decreases the gravitational force and results in sinking rates that are lower. Applying fractal scaling concepts^38^ to describe the particles, and using the same procedure as above (see Supplementary Sec. 2 for a full derivation), we can calculate the mass flux of aggregates as

12 $${F}_{M}^{agg}(z)=\left\{\begin{array}{ll}{N}_{0}{\rho }_{eff}\frac{4}{3}\pi {\left(\frac{1}{{C}_{agg}}{\rm{l}}{\rm{o}}{\rm{g}}\left(1-\frac{\beta }{\alpha }z\right)+{a}_{0}^{D}\right)}^{\frac{4}{3}D} & {\rm{f}}{\rm{o}}{\rm{r}}\ z\le {z}_{dis}\\ 0 & {\rm{f}}{\rm{o}}{\rm{r}}\ z\le {z}_{dis}\end{array}\right.$$

$${\rm{where}}$$

$${C}_{agg}=\frac{2{\rho }_{T}\beta g{A}_{f}{a}_{s}^{3-D}}{9\mu r{a}_{0}D}$$

*D* is the fractal dimension (1.6 < *D* < 3^38^), *A*_*f*_ is a constant^38^ and *a*_*s*_ is the sub-particle radius (Supplementary Fig. S2). It is satisfying to note that the aggregate solution converges to that for a solid particle (eqn 9) as *D* approaches 3 (the fractal dimension of a solid sphere) and as *a*_*s*_ approaches *a*_0_.

Numerous observations of settling rate versus size demonstrate that (particularly for larger aggregate particles) the spectra are flatter than predicted by Stokes’ equation applied to spherical particles^9,27^. In our model, the spectrum flattens as the fractal dimension *D* is reduced below that predicted for a solid particle (D < 3). Here, a fractal dimension of D = 1.85 optimizes the fit between the theoretical settling rate (gray points, Fig. 3a) and the relationship observed^27^ for large aggregates (red dashed line). The modeled sinking rate at *z* = 0 with *α* ranging from 0.01 to 0.1 for small particles (spread in the black points, Fig. 3a) spans a similar range to what is observed (red circles)^29^.

Laboratory-based studies indicate that typical sinking speeds of individual diatom cells range from 0.1–10 m d^−1^ ^42^, and aggregates sink at 88–569 m d^−1^^27^. Using optical proxies, silica-depleted diatom cysts^43^ were observed to be sinking at 75 m d^−1^ during the North Atlantic spring bloom^44^. Plumes of aggregates (>1 mm) were found to be sinking at 45–108 m d^−1^ in the California Current^45^. The size-specific settling rate near the west Antarctica Peninsula^37^ showed large departures from the monotonic increase in settling predicted by Stokes’, potentially due to size-dependent density or shape characteristics.

The excess density of oceanic particles is challenging to measure directly and has generally been inferred from the Stokes’ equation using the sinking velocity, size, and fluid density^29,39,40,46^. McCave (1975)^29^ quantified the sinking rate of small organic particulates from various ocean basins over the size (diameter) range of 1.4 to 362 *μ*m (open circles, Fig. 3a). The sinking rates for these particles ranged from 0.1 to 100 m d^−1^, from which, a excess density ranging between 500 and 29 kg m^−3^ was inferred (open circles, Fig. 3a). Eppley^47^ performed similar tests on living and senescent diatoms, finding an enhanced excess density (and higher sinking rate) for the latter category. In our model, solid particles have a size-independent excess density (black points, Fig. 3b) that has a similar magnitude to the observed values^29^. However, a weakly negative relationship observed between excess density and size^29^ suggests that even small particles may have aggregate-like characteristics^48^. Observations of large aggregates^39,40^ revealed a negatively sloped excess density spectrum (red dashed lines, Fig. 3b) up to two orders of magnitude lower than that predicted for smaller particles (red dashed lines, Fig. 3b). Similarly, the model predicts a negative slope for aggregates, as the volume fraction of particulate material decreases as aggregate size increases.

### Particle compressibility

**Figure 4:**
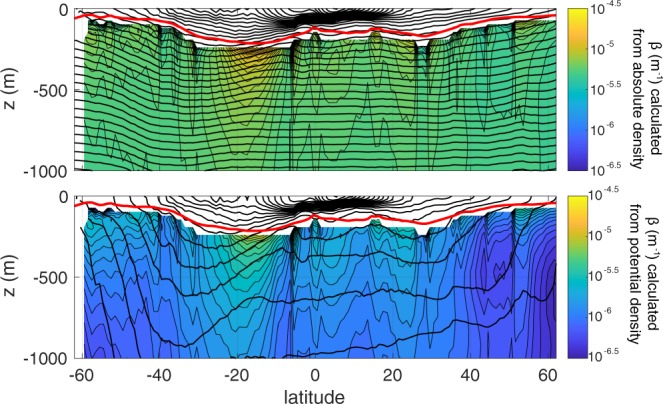
Meridional transects of *β* reflecting (**a**) pressure-driven absolute density, and (**b**) potential density. These values were computed from the WOA09 database from zonal averages between 10.5 to 28.5 ^*o*^W in the Atlantic Ocean. Thick black lines indicate the density contours, and the red line is the mean euphotic depth from a climatological SeaWIFS K490 product. *β* is calculated over over the depth range examined in our model from z_eu_ to 1000 m.

**Figure 5:**
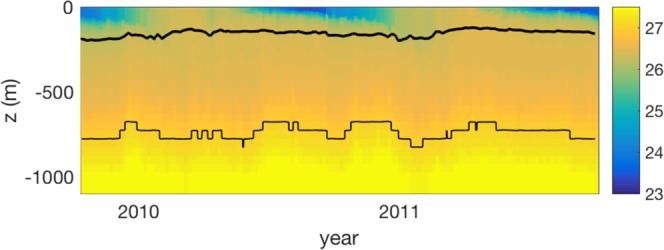
Time-series of potential density -1000 kg m^-3^ (*σ*_*t*_, colors) from ARGO float 6391bermuda (from the chemical sensor group at MBARI www.mbari.org) with contour lines indicating the depth of neutral density for a compressible (thick line) and incompressible (thin line) particle with density *ρ*_*p*_ = 1027 kg m^−3^.

In a stable water column, the absolute density of seawater increases by roughly 6% between the base of the euphotic zone and 1000 m depth, as a result of increasing hydrostatic pressure. In comparison, variation in temperature and salinity with depth cause an increase in density of only 1% to 2% (depending on season and region). For organic marine particles that are only marginally denser than the ambient seawater, variations in the density of seawater have a potentially significant impact on the depth to which a particle will sink. The importance of depth-dependent variations of *ρ*_*f*_ depend, to first order, upon the compressibility of the particles themselves. For incompressible particles, we recommend using a value of *β* that is based on a linear fit between the seawater’s absolute density and depth (see Fig. 4a), whereas for compressible particles, a fit based on potential density may be most appropriate (see Fig. 4b). Overall, we find that most dense particles with (*α* > 0.01) will not strongly ‘feel’ the effect of density, whereas those that have a small *α* may be strongly affected, reaching a suspension depth before being remineralized. In Fig. 5 we illustrate the different fates for an incompressible and compressible particle with a density only slightly greater (*α* = 0.0004) than the density at the base of the euphotic zone (*ρ*_*T*_). In this case, the incompressible particle will reach a neutral density at a much shallower depth (thick black line, Fig. 5) than the depth reached by particles with a similar compressibility as seawater (thin black line, Fig. 5).

### Functional form of particle remineralization

**Figure 6:**
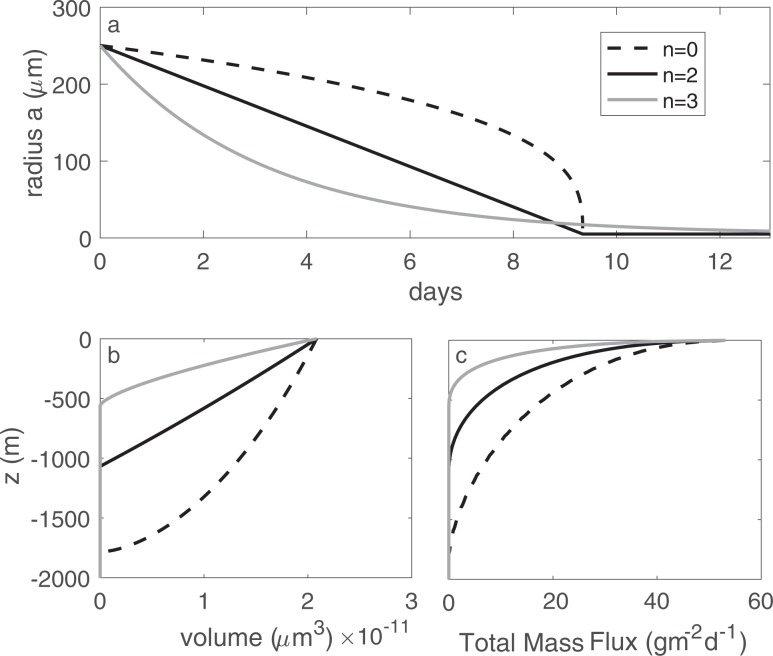
(**a**) The change in size of a particle of initial radius *a*_*o*,*i*_ = 250 *μ*m, *r* = 0.11 d^−1^, as it is consumed according to eqn (2) for the three remineralization cases *n* = 0, *n* = 2 and *n* = 3. (**b**) The change in volume as a function of depth for a single size class of particles with *a*_*o*,*i*_ = 250 *μ*m. (**c**) The resulting flux profile obtained from integrating over a range of size classes with a size spectral slope of −3, as in Fig. 2.

The generalized form for particle volume loss (remineralization) is given in equation (2), wherein the value of *n* determines whether remineralization occurs (a) at a constant rate (*n* = 0), suggesting that a fixed number of bacteria colonize the particle irrespective of particle size, (b) proportional to the surface area (*n* = 2), or (c) proportional to the particle volume (or mass) (*n* = 3). To be dimensionally consistent, the constant *C*_*n*_ takes the form ${C}_{0}={a}_{o}^{3}/3$, *C*_2_ = *a*_*o*_ and *C*_3_ = 3 for *n* = 0, 2, 3, respectively. Here, magnitudes for *C* and *r* were selected to produce an overall life-span of about 10 days (Fig. 6a). These three cases produce markedly different behavior over time: particle size (*a*) diminishes linearly for *n* = 2 (solid black line Fig. 6a), at an increasing rate with constant remineralization (*n* = 0, dashed line Fig. 6a), and at a slowing rate with *n* = 3 (gray line Fig. 6a). Since sinking speed is proportional to *a*^2^, it means that although the life-span of each particle case is similar, particles that are initially consumed more rapidly (*n* = 3) do not sink as deep as particles that that are consumed steadily (*n* = 2) (Fig. 6b). Thus, while the overall shape of the flux profile retains a Martin-like shape (Fig. 6c), it appears that the functional form of remineralization actually impacts the efficiency of transfer.

### Sensitivity of the flux profile to particle density, slope of the particle size spectrum, remineralization rate and oceanic density stratification

**Figure 7:**
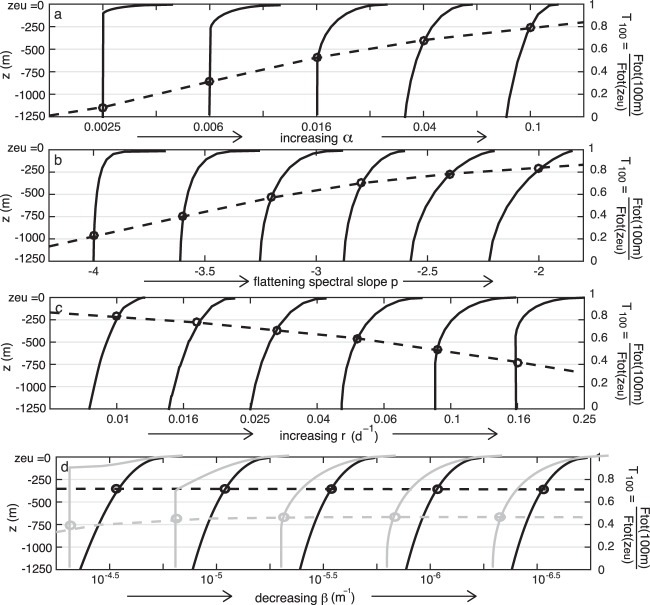
Variety of flux profiles (solid lines) that are obtained from Equations 10 and 11 spanning the observed ranges of (**a**) excess particle density factor *α*, (**b**) initial size spectral slope *p*, (**c**) remineralization rate *r* and (**d**) vertical density change *β*. In each case, one parameter is varied, and the others are kept fixed at *α* = 0.03, *p* = − 3, *r* = 0.05 d^−1^ and *β* = 5 × 10^−6^ m^−1^ (black lines). For panel (**d**) we overlay (in gray) flux profiles obtained with *α* = 0.005 to demonstrate that the profiles are sensitive to *β* when *α* is very small. The transfer efficiency *T*_100_ is shown with open circles intersecting each profile, with its magnitude defined by the axis on the right hand side of the plots.

To assess the sensitivity of the modeled flux profile to our remaining parameters: particle density (*α*), size spectral slope (*p*) and remineralization rate (*r*), we generated 27 flux profiles for which we vary one parameter while holding the others constant. For each profile, we quantify the transfer efficiency, *T*_100_, the ratio of the mass flux at the base of the euphotic zone (*z* = 0) to the mass flux 100 m below the euphotic zone (*z* = 100m)^50^

13 $${T}_{100}=\frac{{F}_{tot}(z=100\ m)}{{F}_{tot}(z=0)}$$

shown with open circles connected by a dashed line in Fig. 7. The range of parameter values were selected from published field and laboratory observations (Table 1), and produce a variety of flux profile shapes and efficiencies (Fig. 7). We fit (*fit*) each of the modeled (*mod*) curves (by minimizing the rms error) with a power law (Martin curve) fit *F*(*z*) = *F*(100*m*) (*z* /100*m*)^*b*^ and an exponential fit *F*(*z*) = *F*(0*m*) *exp*(− *z*/z^*^). The mean rms error is defined as

14 $$erro{r}_{rms}=\left\langle \frac{{(mod-fit)}^{2}}{mo{d}^{2}}\right\rangle $$

We find that 11 (of 27) cases modeled are better fit by an exponential, 10 are better fit by a power law, and for the remaining 6, neither the exponential nor power law fit is distinguishably better. Essentially, neither a power law nor exponential form is clearly better at describing these profiles. We had hoped that in performing this test, we could shed light or which is the more suitable form to use for empirical data fitting, but the answer remains elusive. In the following sections, we examine the role that each of the model parameters plays in shaping this curve.

#### Particle density

The excess density of oceanic particles is challenging to measure directly and has typically been inferred from the Stokes’ equation using the sinking velocity, size, and fluid density^29,39,40,46^. McCave (1975)^29^ quantified the sinking rate of small organic particulates from various ocean basins over the size (diameter) range of 1.4 to 362 *μ*m. The sinking rates for these particles ranged from 0.1 to 100 m d^−1^, from which, an excess density ranging between 500 and 29 kg m^−3^ was inferred. Eppley *et al*.^47^ performed similar tests on living and senescent diatoms, finding an enhanced excess density (and higher sinking rate) for the latter category.

As anticipated, increasing the excess particle density factor *α* results in an increased efficiency of export (open circles, Fig. 7a), as denser particles sink faster, are less affected by changes in the ambient fluid density *β*, and reach greater depths before they are remineralized. A similar result was obtained by Cram *et al*.^13^ for a larger percentage of ballast in particles. Over the modeled range of *α* = (0.002–0.1), between 8% and 80% of the exported material escapes consumption within *z* = 100 m according to the model.

#### Slope of the particle size spectrum

Flattening the initial size spectrum of *a*_*o*,*i*_ and transitioning the mass spectrum from one dominated by small particles (*p* < −3) to one dominated by large particles (*p* > −3) also increases the transfer efficiency (Fig. 7b) as in Cram *et al*.^13^. Under our assumption that particles are remineralized at a steady rate, this makes intuitive sense. As with increasing *α*, larger particles sink more rapidly and reach greater depths before they are remineralized. The model predicts that *T*_100_ could vary between 0.2 and 0.84 for variations in *p* between −4 and −2 throughout the global ocean^19^.

#### Remineralization rate

As predicted in other export models^51^, we find that increasing remineralization rate over a range of observed values (*r* = 0.01–0.16 d^−1^) attenuates the flux profile and decreases the export efficiency *T*_100_ from 0.82 to 0.4 (Fig. 7c). We also find that the functional form selected for *r* (equation 2) can alter the depth to which a particle is likely to sink (Fig. 6b) and transfer efficiency (Fig. 6c). In the Discussion, we evaluate the different circumstances under which one might choose a particular functional form over another. A model that explicitly includes a temperature-dependent^13,28,52^ or oxygen-dependant^13^ *r* could allow more material to sink to depth than is predicted by our model. Additionally, variability in *r* may arise from losses and transformations of POC through zooplankton feeding and egestion^53–55^ and from the varied composition of particles.

#### Water column density change

As a particle sinks it encounters denser water, the relative density between water and particle is reduced, the buoyancy force on the particle increases, and the particle may slow down or become suspended. While the effect of density stratification on sinking marine snow or pellets has been considered in other literature^56,57^, mechanistic flux models^10,12,13^ have not included vertical density changes. The parameter *β* approximates the linear change in water column density with depth according to (4). Since this parameter is not reported in previous literature, we calculate it explicitly from World Ocean Atlas data across a meridional transect between the climatological euphotic depth (red line, Fig. 4) and 1000 m. We consider *β* calculated from the absolute density (Fig. 4a), as would be appropriate for incompressible particles (see the earlier section on ‘Particle compressibility’), and *β* calculated from the potential density (Fig. 4b), as appropriate for compressible particles. Across this transect, *β* varies from about 5 × 10^−4^ to 5 × 10^−6^. The effect of *β* is pronounced for particle densities that are only slightly greater than *ρ*_*T*_ (i.e. *α* = 0.005, gray lines Fig. 7d). However, for perhaps a more typical value of *α* = 0.03, the overall flux profile is not very sensitive to the choice of *β* (Fig. 7d). Interestingly, for the low *α* case, though the overall profile shape (solid gray lines) is strongly altered by *β*, this sensitivity is not reflected in the transfer efficiency, which remains between 0.4 and 0.5 for each of the five cases considered (dashed gray line). This is because *β* has the largest impact when the particles reach a suspension depth (as is the case for the first two gray profiles, *β* = 10^−4.5^ and 10^−5^ m^−1^ on Fig. 7d), but does not appear to strongly alter the shape of the profile above that depth.

### Evolution of the particle size spectrum with depth

**Figure 8:**
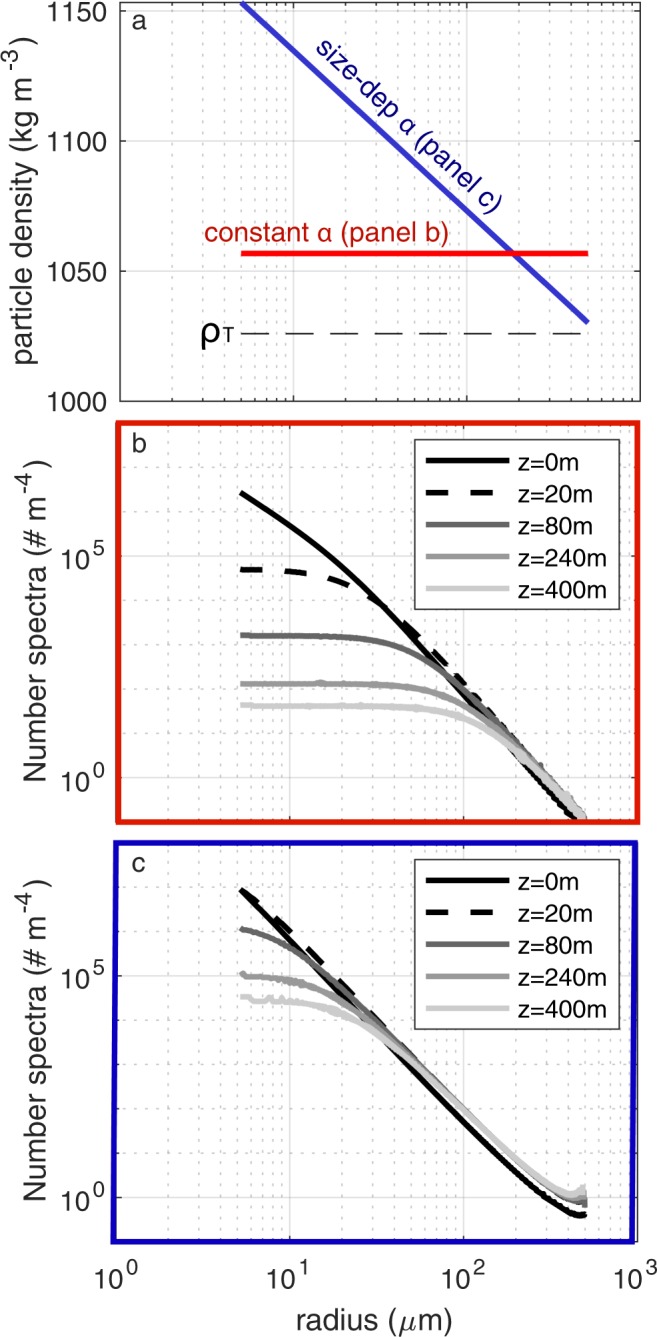
A size-dependant particle density can be used to counteract the flattening effect seen with depth in the model. (**a**) The excess density that results from a constant *α* = 0.03 (blue line) and *α* that linearly decreases from 0.125 to 0.004 over the specified log-distribution of particle sizes (red line). (**b**) The size spectrum is shown to flatten with depth in the constant *α* case. (**c**) With a size-dependant *α*, this effect is markedly reduced. A similar result can be achieved with an *r* that increases with increasing particle size (not shown).

The model described here, as well as the models of Kriest and Oschiles^12^ and De Vries *et al*.^10^, predict that the size spectrum of particles will flatten with increasing depth in the water column, as smaller particles are lost through remineralization and not replenished in large enough numbers by the remineralization of larger particles, of which there are relatively few (Supplementary Sec. 1). However, there is no hint of such a flattening of size spectra in observational data^19^. Several reasons can be postulated for this: (i) A large particle undergoes disaggregation or break up into many smaller particles during its descent; (ii) Particles contain hard, mineralogical or recalcitrant bits, so that the remineralization rate diminishes with particle size as the softer material is eaten away; (iii) the net particle density increases with diminishing particle size for the same reason. While a disaggregation model is beyond the scope of this study, we show that by decreasing particle density (Fig. 8) or increasing *r* (not shown) with particle size, the slope of the size spectrum can be largely maintained with depth. Fig. (8a) provides a demonstration of how size spectra may be altered with depth as a result of a linear dependence between *α* and size. The range in *α* selected here is based on the observed ranges cited in Table 1, and the hypothesis that small particles are likely to be more dense (with more ballast) than their larger, more fluffy counterparts^17^. This approach provides a plausible remedy to the inconsistency of our original model, as well as other models^10,12^.

### Modeling a transient phytoplankton bloom by varying the particle size spectrum

A transient phytoplankton bloom is simulated by steepening the size-spectral slope from *p* = −2 to *p* = −4.5 over 3 days to represent the transition from early bloom, with production of more large particles (diatoms) to late bloom, which is dominated by smaller particles. Such a scenario was observed during the North Atlantic Bloom experiment, where diatoms dominated the early bloom, but were superseded by smaller phytoplankton when silica ran out^44,58^.

For the transient solution, we are no longer able to use the analytic solution and, instead, implement the model numerically. Initially, the slope of the particle size spectrum is set to *p* = −2. The model is integrated for 16 days until the solution reaches a steady state. From day 16–19, the size-spectral slope is changed linearly from *p* = −2 to *p* = − 4.5. After day 19, *p* is held constant until the new steady state is reached. As *p* is varied, the total mass flux leaving the base of the euphotic zone is held constant. This ensures that only the relative numbers of large and small particles are varied without introducing changes to the total mass flux entering the water column.

#### Numerical model

We use a Lagrangian model based on Jokulsdottir and Archer^11^ to compute the time-dependent solution. A particle object is used to represent a certain number of particles with identical properties, such as size and density, when released from the base of the euphotic layer. Particle objects can be created at each time step, and their trajectories are found by numerically integrating Equation (5) using the Forward Euler method and interpolating the solution onto a grid with 0.5 m resolution. A time step of 1 hour yields a percent error <0.4% when comparing the analytical and numerical solutions for a 250 *μ*m particle, sinking over the course of 20 days. The mass flux at each time is calculated as *F*_*m*_(*t*) = *N*_0_*ρ*_*p*_*V*(*t*) where $V(t)=\frac{4}{3}\pi {[{a}_{0}(1-rt)]}^{3}$ for the remineralization case *n* = 2. The total mass flux is a function of depth and time and is obtained by summing all the particle objects. For the transient bloom simulation, ten particle objects are created at each time step with size classes ranging from 25 to 250 *μ*m. The values of *α* = 0.03, *r* = 0.05 d^−1^, and *β* = 5 × 10^−6^ m^−1^ are used for all particle objects.

#### Results of numerical model

**Figure 9:**
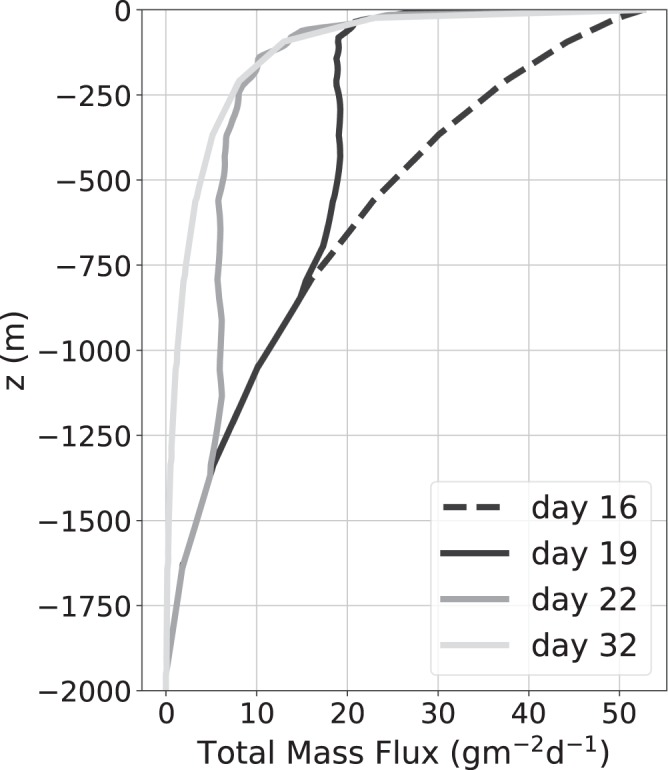
Time evolution of the vertical flux profile as the slope of the particle size spectrum is varied linearly in time from *p* = −2 on day 16 to *p* = −4.5 on day 19, after which it is held constant. The total mass flux exiting the euphotic layer is held constant and other parameters are fixed at *α* = 0.03, *r* = 0.05 d^−1^, and *β* = 5 × 10^−6^ m^−1^.

 Figure 9 shows the total mass flux profiles at four different times of the simulated transient bloom. The profile on day 16 (dashed line) is the initial steady state profile for *p* = −2, which represents a relative abundance of large particles. The final steady state profile for *p* = −4.5 corresponding to a relative abundance of small particles is reached by day 32 (light gray line). Profiles between days 16 and 32 show the transition between these two steady states as a result of varying the size-spectral slope at the base of the euphotic layer from day 16 to 19. A subsurface mass flux peak is seen in Fig. 9 on day 19 (black solid line) at a depth of *z* = 500 m. This subsurface bump then propagates down through the water column with time, reaching a depth of around *z* = 1250 m by day 22 (dark gray curve in Fig. 9). Note that the total mass flux entering the water column is held constant, so these subsurface peaks are solely the result of changing *p* at *z* = 0 m.

**Figure 10:**
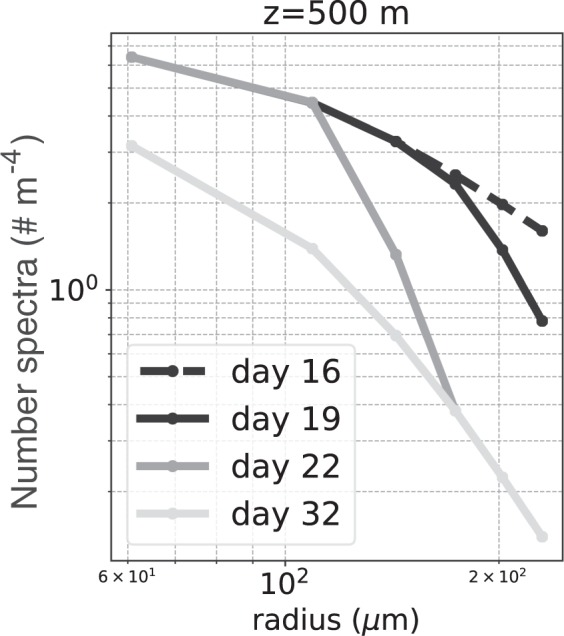
Time-evolution of the particle size spectrum (PSD) at *z* = 500 m as the number spectral slope at the surface was varied from *p* = −2 to *p* = −4.5. The largest particles transition more rapidly to the new steady-state, and the smallest particles transition more slowly.

The particle size spectra (PSD) at a depth of 500 m (Fig. 10) reveal that the bump in the flux profile (Fig. 9) is caused by a relative abundance of large particles compared to small particles at the base of the euphotic layer. On day 19, the size spectrum at *z* = 0 m is steepened so there is a reduction in the number of large particles entering the water column. However, it takes time for this information to reach depth, so the size spectrum at *z* = 500 m is still relatively flat, and there are more larger particles at *z* = 500 m than at *z* = 0 m on day 19. This excess of large particles at depth creates the subsurface maxima in mass flux seen in Fig. 9. Similarly on day 22, the excess of large particles at *z* = 1250 m results in another subsurface maxima in mass flux.

Furthermore, the numerical model reveals how the transition between two steady states occurs. Because larger particles sink faster in this model, the transition between two steady-state spectra occurs starting with the largest particles. For example, Fig. 10 shows the time-evolution of the size spectrum at *z* = 500 m. As *p* is steepened at *z* = 0 m, the spectrum at *z* = 500 m steepens starting from the largest particle sizes. By day 22, the largest three particle sizes have already reached the new steady-state spectrum. In contrast, the smaller particles do not reach the new steady-state spectrum until day 32. This is because the small particles sink more slowly, so it takes longer for information about changes in small particles at the surface to reach depth.

## Discussion

There have been several attempts^10,12^ to mechanistically reproduce the canonical Martin flux profile from first principles using only a few parameters. Here, we derive the flux profile based on four parameters: the excess particle density *α*, the change in seawater density with depth *β*, remineralization rate *r*, and spectral slope of the particle size distribution *p*. We examined the influence of each of these parameters over a range of realistic values derived from literature (Table 1) and/or observations (Fig. 4). Most Stokes’-derived analyses of sinking oceanic particles to date have not accounted for the change in the density of seawater with depth, despite some observational evidence that it may be of importance for certain particle types^59^. Our work suggests that *β* becomes increasingly important in cases where $\frac{\beta }{\alpha }z > 1$, and appears to be disproportionately important for porous particle aggregates that are permeated by the surrounding water (and differ from those presented here). Some field and laboratory based observations support this premise. For example, accumulation of marine snow of moderate to high porosity is observed^60^ at high density gradients in the water column. More recently, it was pointed out^61^ that particle accumulation at density interfaces may be enhanced by the diffusion-limited exchange of low density water entrained within aggregates. For incompressible particles, we recommend using a value of *β* derived from linear fits to the absolute water density, which is relatively constant throughout different regions and seasons (Fig. 4a). For particles with a compressibility similar to that of seawater, using *β* derived from a linear fit to the potential density of seawater would be more appropriate (Fig. 4b).

There is presently no clear consensus on the best model for remineralization. We offered a generalized form (eqn 2), and applied the surface-area dependent case (*n *= 2) for the analysis. Experiments by Smriga *et al*.^23^ suggest an area-dependent remineralization rate that can become volume-dependent if particles are colonized internally. Iversen *et al*.^27,28^ demonstrated that aggregates with equivalent spherical diameters of 1 to 5 mm have a total respiration rate (measured in mol O_2_ produced per *μ*gC by aggregates in each size class) that is linearly related to particle mass. These observations imply that remineralization modeled with *n* = 3 could be most appropriate for aggregates, whose porous shapes allow for colonization throughout their volume, provided that the mass of the aggregate is linearly related to its volume. An exception to this case may arise if the aggregates are internally anoxic^62^, in which case, surface area-dependency (*n* = 2) may become more appropriate. A number of observational^27,40^ and theoretical^63^ papers suggest that the volume-mass relationship in aggregates is nonlinear, and could be modeled with fractals. Also, the mode and rate of shrinkage could change in time as the more labile organic matter is consumed and the proportion of recalcitrant material increases. In general, shrinkage of particles would lead to a slowing down of the sinking velocity according to Stokes’ law, but there have been examples that challenge even this premise^64^, implying that a diminishing fraction of labile to lithogenic material may represent an increase in particle density *ρ*_*p*_ as the particle ages.

We compared our modeled sinking rates *w*_*sink*_ with those reported in the literature. Laboratory-based studies indicate that typical sinking speeds of individual diatom cells range from 0.1−10 m d^−1^^42^, and aggregates sink at 88–569 m d^−1^^27^. Using optical proxies, silica-depleted diatom cysts^43^ were observed to be among the particles sinking at ~ 75 m d^−1^ during the North Atlantic spring bloom^44^. Another estimate of settling velocities^29^ found they varied from 0.57 to 105 m d^−1^ over the range of particle sizes that we model here. An explicit test of our model is not possible without also knowing the excess particle density and mass-volume relationship of the particles in these studies. However, the observed sinking rates overlap, and are generally centered within the range of 0.1 to 500 m d^−1^ predicted from (5) using the range of parameters listed in Table 1. We emphasize, however, that there are significant limitations in using Stokes’ law across a broad range of particle types. For example, Mcdonnell and Buesseler^37^ showed significant departures from the monotonic increase in settling speed predicted by Stokes, potentially due to size-dependent density or shape characteristics.

Our model assumes that the total flux can be written as a linear sum of the flux across a range of size classes – a scenario that assumes particles do not collide, coagulate, or fragment as they sink. Interactions between particles (particularly at high particle concentration), and aggregation due to stickiness may depend on the particle composition, cells’ physiological state, microbial environment, and other factors that are not modeled here^65^, but reviewed in the literature^63^. Thus, we suggest that the approach described here is most appropriate in conditions of low particle concentration with low probability of collisions.

In conclusion, our analytic model, based on a small set of parameters, generates a range of shapes in the flux profile (Fig. 7). In spite of its underlying assumptions and lack of complexity, the model is illustrative of some principles. A single type and size of particle cannot produce the canonical flux profile, but summing over an allometric size distribution (or a range of particle densities or characteristics, not shown) achieves the characteristic Martin curve shape. We find that neither a power law, nor an exponential can, by itself, describe the entire gamut of flux profile shapes obtained from varying the few parameters in the model. We also demonstrate the impact that time-dependence arising from a community transition, such as an abrupt fall-out after a large diatom bloom, has on the profile shape. Though the model neglects factors like aggregation^63^, disintegration, ballasting by minerals^8^, particle shape, and temperature-dependent remineralization^52^, it is able to capture a wide range of flux profile shapes and characteristics. Such a model provides a useful framework for examining the relative importance of particle and environmental parameters to the flux, and provides guidance on how to allocate observational efforts to measuring relevant characteristics that shape the export flux profile.

## Supplementary information

Supplementary Information.
