# Supplementary material for: Sinking flux of particulate organic matter in the oceans: Sensitivity to particle characteristics
Source: Sci Rep. 2020 Mar 27;10:5582. doi: 10.1038/s41598-020-60424-5 (PMC7101343; doi:10.1038/s41598-020-60424-5)
Supplement: Supplementary file 1 — Supplementary Information. [file 41598_2020_60424_MOESM1_ESM.pdf]

Supplementary Material
Sinking flux of particulate organic matter in the oceans: Sensitivity to particle characteristics

Melissa M. Omand[1], Rama Govindarajan[2], Jing He[3], and Amala Mahadevan[4*]

[1]University of Rhode Island, Graduate School of Oceanography, Narragansett, RI 02882, USA
[2]International Centre for Theoretical Sciences, Tata Institute of Fundamental Research, Bengaluru 560089, India
[3]MIT/WHOI Joint Program in Oceanography, Woods Hole, MA 02543, USA
[4]Woods Hole Oceanographic Institution, Woods Hole, MA 02543, USA
[*]amala@whoi.edu

1 Modeling the particle size distribution with depth

Below the euphotic layer, we assume that turbulence is weak and does not alter the particle's descent rate. In a turbulent ocean, the molecular viscosity, used in our model, may be replaced by the eddy viscosity. Convective deepening of the mixed layer[1,2] and advection by currents[3] can contribute to passive particle export, but here we focus only upon the sinking fraction, and assume that beneath $z = 0$ (the euphotic base and top of our model domain), particle transport is dominated by sinking.

One of the more prominent, and potentially problematic, predictions by the model is that the size spectral slope of the particles flattens with depth (Fig. S1ab). The smallest initial size classes are remineralized nearer the surface, and the larger sizes transition to smaller-size categories. The spectrum of mass flux (SI, Fig. S1cd) for the case with a number spectral slope $p = -3$ is initially fairly flat and steepens with depth as the particles that started small disappear altogether, and the particles that started large become smaller. Similar models (PRISM,[4]) that use a Stokes-based sinking rate also predict a flattening of the spectrum, however the observational evidence does not support this[5,6]. Recent approaches have tried to reconcile this by incorporating a dissaggregation term to restore the small particle class[7]. However it is also possible that small particles are persistent at depth because they are sinking more rapidly due to a hitherto unexplained mechanism such as different ballast or fractal dimension than larger aggregate particles, or they are less labile and persist for longer than their larger counterparts. If further observational evidence were discovered to inform this, the model could be adapted to have a disaggregation term, or a size-dependant density (ballast) or remineralization term. In that case, the analytic solutions presented here would not hold, and the solution would likely need to be obtained numerically. Some progress is made to this end by modeling the particles as aggregrates. In this case, a reduced fractal dimension means that larger particles are 'fluffier' and the drag force relative to their mass is enhanced. This has the effect of reducing the flattening trend shown in Fig. S1. The full derivation of this solution is given below.

2 Modeling aggregates

In this theory, we use the fractal dimension, D, to represent the geometry of particles that are aggregates of material. Particle aggregates are most likely to form within the euphotic zone, where particle abundances are high, and the probability of particle collisions is enhanced through elevated shear and turbulence within the mixed layer[8], by coagulation, or by the repackaging of organic material through trophic interactions, ingestion and egestion.[9] Aggregates, whose effective radius we denote by a_g, are typically large particles composed of many loosely or tightly packed sub-particles (Fig. S2). They have a size-dependent volume to mass relationship that can be parameterized according to the particle fractal dimension, D[10], that typically varies between 1 and 3. Fluffier, more porous aggregates have low values of D, while compact aggregates have a value of D that approaches 3 (the limit for a solid sphere).

Aggregates formed by Brownian motion, shear coagulation and differential sedimentation have fractal geometry[9]. This property allows a scaling between the fraction of sub-particles and the particle size $f = A_f(\frac{a_g}{a_s})^{D-3}$, introducing two new variables; the fractal dimension D (must be < 3) and a constant A_f[10]. Over the range of reported values of D for different aggregation experiments[11] we find that our results are not sensitive to this choice. Thus, we can write the Stokes sinking velocity for an aggregate and proceed in a similar manner as the solution given above.

An aggregate of radius a_g is composed of many sub-particles, here assumed to be all of radius a_s and density ρ_p. We

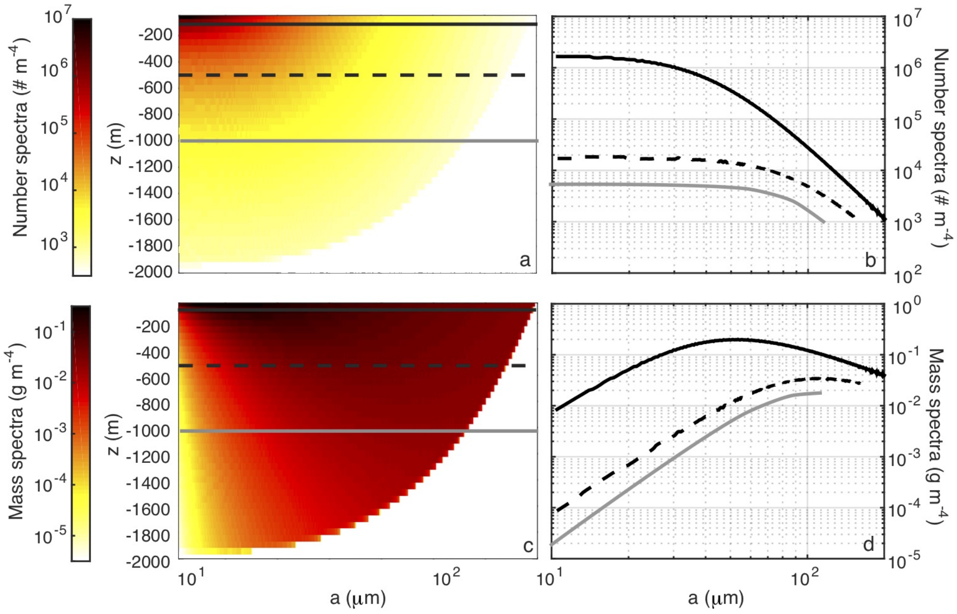

Figure S1. The a) number density and c) mass density of sinking particles predicted from (10) and (11) in the main text, with an initial ($a_{o,i}$) number spectral slope $p = -3$, $r = 0.05\ \mathrm{d}^{-1}$, $\alpha = 0.03$ and $\beta = 5 \times 10^{-6}$. The trajectory of each $a_{o,i}$ is shown in a) by following the colored isolines downwards, passing through diminishing size classes until intersecting the vertical axis at $z_{dis}(a_{o,i})$. b) The number spectrum predicted by our model at $z = 100$ m (black solid line), $z = 500$ m (black dashed line), and $z = 1000$ m (gray line). Overall, the size spectral slope flattens with depth, and the small particles present at depth represent those that began in a larger size category. d) The mass spectrum predicted by our model at $z = 100$ m (black solid line), $z = 500$ m (black dashed line), and $z = 1000$ m (gray line). The spectral mass density for this distribution of a_0 is roughly flat near the surface, and steepens with depth as the largest particles constitute an increasing fraction of the total mass.

now have a permeable particle sinking at velocity w, on which the drag force is reduced by a factor Ω given by Debye and Brinkman[12] as

$$\Omega = \frac{2\beta^2\left[1 - \frac{\tanh\beta}{\beta}\right]}{2\beta^2 + 3\left[1 - \frac{\tanh\beta}{\beta}\right]}, \tag{1}$$

where β^{-2} is the non-dimensional permeability. The drag force on an aggregate is thus given by

$$\mathrm{F_d} = 6\Omega\pi\mu a_g w. \tag{2}$$

Here we assume that although an aggregate has a large porosity[13], the permeability (flow of water through the aggregate) is low, so $\beta \gg 1$ and $\Omega \simeq 1$. An aggregate of radius a_g is composed of many sub-particles, here assumed to be all of radius a_s and density ρ_p.

If the aggregate were compressed into a solid ball, it would have a radius a and a gravity force F_g. But, F_d can also be written with an effective density ρ_{eff} as though the particle has diameter a_g and its density is reduced because a fraction $(1-f)$ of the particle volume is filled with the ambient fluid (we also neglect that the aggregate could entrain less dense water downward along with it, as it sinks[14]).

The gravitational force on the particle is therefore given by

$$\mathrm{F_g} = \frac{4\pi}{3}a^3 g(\rho_p - \rho_f) = \frac{4\pi}{3}a_g^3 g(\rho_{eff} - \rho_f), \tag{3}$$

where ρ_{eff} is

$$\rho_{eff} = f\rho_p + (1-f)\rho_f. \tag{4}$$

Jackson[10] relates a to a_g according to

$$\left[\frac{a}{a_s}\right]^3 = A_f\left[\frac{a_g}{a_s}\right]^D, \tag{5}$$

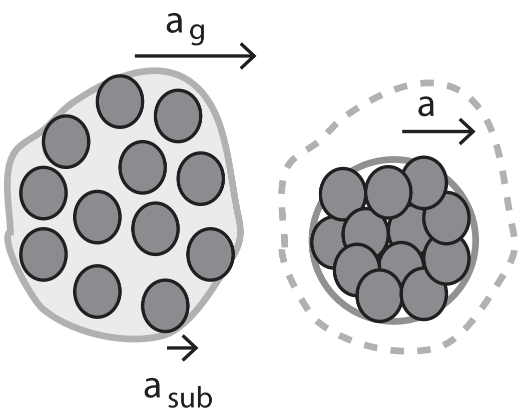

Figure S2. Conceptual schematic of an aggregate with radius a_g composed of sub-particles of radius a_s and density ρ_p. If the aggregate were to be compacted into a solid ball, the new radius would be a and the fraction of the new volume relative to the initial volume, f. The 'effective density' of the aggregate is therefore $\rho_{eff} = f\rho_p + (1-f)\rho_f$.

where A_f and D are empirical constants (Jackson[10] uses $A_f = 1.49$, $D = 2.33$). D is the 'fractal dimension' (for a solid object, D=3). So, the volume fraction (f) becomes

$$f = A_f \left[\frac{a_g}{a_s}\right]^{D-3}. \tag{6}$$

Substituting f into (3) and (4) we get

$$F_g = \frac{4\pi}{3} g A_f \left[\frac{1}{a_s}\right]^{D-3} (\rho_p - \rho_f)\, a_g^D \tag{7}$$

Equating the two forces and solving for the terminal velocity of the aggregate

$$w_{agg} = \frac{dz}{dt} = \frac{g A_f (\rho_p - \rho_f)\, a_g^{D-1}}{18\,\mu\, a_s^{D-3}} \tag{8}$$

Equation (8) is integrable by separation of parts and is combined with remineralization $a_g = a_0(1-rt)$ to arrive at the solution for the aggregate radius a_g as a function of depth,

$$a_g = \left[\frac{1}{C_g} log \left[\frac{1-\frac{\beta}{\alpha}z}{1-\frac{\beta}{\alpha}z_{eu}}\right] + a_0^D\right]^{1/D}, \tag{9}$$

where

$$C_g = \frac{2\rho_T\,\beta\, g A_f\, a_s^{3-D}}{9\,\mu\, r a_0 D}. \tag{10}$$

The void space within the particles must be accounted when computing the mass flux $F_{M\,agg}$ for aggregates with a range of sizes. This can be accomplished be multiplying the volume $V_g^n = \frac{4\pi a_g^3}{3}$ of each size class n by its 'effective' density ρ_{eff}. Thus, for N^n particles of size class n, the mass flux (per m^2 per day) is given by

$$F_{M\,agg}^n(z) = \begin{cases} \rho_{eff}^n N^n V_g^n(z), & \text{for } z_{eu} \le z_d \le z_{p\,dis} \\ 0, & \text{for } z_d > z_{p\,dis}. \end{cases} \tag{11}$$

The total mass flux is again the sum over all size classes and is given by

$$F_{tot\,agg}|_z = \sum_{n=1}^{n_{max}} F_{M\,agg}^n|_z. \tag{12}$$

References

1. Giering, S. *et al.* High export via small particles before the onset of the North Atlantic spring bloom. *J. Geophys. Res.* (2016).
2. Koeve, W., Pollehne, F., Oschlies, A. & Zeitzschel, B. Storm-induced convective export of organic matter during spring in the north-east atlantic ocean. *Deep. Sea Res. Part I* **49**, 1431–1444 (2002).
3. Omand, M. *et al.* Eddy-driven subduction exports particulate organic carbon from the spring bloom. *Sci.* **348**, 222–225 (2015).
4. DeVries, T., Holzer, M. & Primeau, F. Recent increase in oceanic carbon uptake driven by weaker upper-ocean overturning. *Nat.* **542**, 215–218 (2017).
5. Laurenceau-Cornec, E. *et al.* The relative importance of phytoplankton aggregates and zooplankton fecal pellets to carbon export: insights from free-drifting sediment trap deployments in naturally iron-fertilised waters near the Kerguelen Plateau. *Biogeosciences* 1007–1027 (2015).
6. Guidi, L. *et al.* Effects of phytoplankton community on production, size and export of large aggregates: A world-ocean analysis. *Limnol. Oceanogr.* **54**, 1951–1963 (2009).
7. Bianchi, D., Weber, T. S., Kiko, R. & Deutsch, C. Global niche of marine anaerobic metabolisms expanded by particle microenvironments. *Nat. Geosci.* **11**, 263 (2018).
8. Hill, P. S., Nowell, A. R. M. & Jumars, P. A. Encounter rate by turbulent shear of particles similar in diameter to the kolmogorov scale. *J. Exp. Mar. Biol. Ecol.* **50**, 643–688 (1967).
9. Shaefer, D. W., Martin, J. E., Wiltzius, P. & Cannell, D. S. Fractal dimensions of aggregates determined from steady-state size distributions. *Phys. Rev. Lett.* **52**, 2371–2374 (1984).
10. Jackson, G. Comparing observed changes in particle size spectra with those predicted using coagulation theory. *Deep. Sea Res. Part II* **42**, 159–184 (1995).
11. Jiang, Q. & Logan, B. E. Fractal dimensions of aggregates determined from steady-state size distributions. *Environ. Sci. Technol.* **25**, 2031–2038 (1991).
12. Neal, G. & Epstein, N. Creeping flow relative to permeable spheres. *Chem. Eng. Sci.* **28**, 1865–1874 (1973).
13. Alldredge, A. L. & Gotschalk, C. In situ settling behavior of marine snow. *Limnol. Oceanogr.* **33**, 339–351 (1988).
14. Prairie, J. C. *et al.* Delayed settling of marine snow at sharp density transitions driven by fluid entrainment and diffusion-limited retention. *Mar. Ecol. Prog. Ser.* **487**, 185–200 (2013).
